# Abstract of Meteorological Observations for July, 1856, Made at Philadelphia, Pa.

**Published:** 1856-09

**Authors:** James A. Kirkpatrick


*Abstract of Meteorological Observations for July*, 1856, *made at Philadelphia, Pa. Latitude* 39° 57' 28" *N., Longitude* 75° 10' 40" *W. from Greenwich.* By Prof. James A. Kirkpatrick.

| 1856. July. | Barometer. Daily Mean | Barometer. Mean Daily Range. | Thermometer. Daily Mean | Thermometer. Mean Daily Range | Thermometer. Extreme Range | Solar Radiation. 2 P.M | Rel. Humid. 2 P.M. | Force of Vapor 2 P.M. | Dew Point 2 P.M. | Rain | Prevailing Winds. | Remarks. |
|---|---|---|---|---|---|---|---|---|---|---|---|---|
| | Inches. | Inches. | Deg. | Deg. | Deg. | Deg. | Hunds. | Inches. | Deg. | Inches. | Points. | |
| 1 | 29.938 | .339 | 78.0 | 12.5 | 13.0 | 50.0 | 28 | .327 | 47.3 | | NW. | Clear. |
| 2 | 29.956 | .059 | 75.8 | 2.5 | 10.0 | 14.0 | 44 | .450 | 56.0 | | (Var.) | Cloudy. *Barometer highest* 29.998. |
| 3 | 29.737 | .219 | 80.0 | 4.2 | 13.5 | 25.5 | 50 | .608 | 64.6 | | SW. | Cloudy. |
| 4 | 29.608 | .128 | 78.2 | 2.8 | 10.0 | 26.0 | 50 | ·582 | 63.3 | | (Var.) | Morning and aft. cloudy; 12 M. until 2 P.M. a few drops of *rain*; evening clear. |
| 5 | 29.707 | .099 | 77.0 | 2.5 | 18.0 | 44.0 | 32 | .354 | 49.5 | | E. | Clear. |
| 6 | 29.667 | .041 | 79.0 | 2.7 | 12.0 | 8.0 | 38 | .478 | 57.8 | 0.054 | SW. | Morning clear; aft. cloudy; 5½ P.M. thunder, 5¾ *rain* until 7¾. |
| 7 | 29.758 | .092 | 77.5 | 2.8 | 14.0 | 28.0 | 56 | .635 | 65.8 | 0.036 | NE. | Cloudy; 5, P.M. until 5¼, *rain.* |
| 8 | 29.814 | .056 | 76.7 | 3.2 | 8.0 | 3.0 | 59 | .623 | 65.3 | 0.182 | SE. | Cloudy; morning and afternoon *drizzling rain.* |
| 9 | 29.841 | .027 | 73.2 | 2.8 | 9.0 | 16 0 | 69 | .638 | 66.0 | | (Var.) | Morning and aft. cloudy; evening clear. |
| 10 | 29.881 | .045 | 73.7 | 3.2 | 13.0 | 47.0 | 58 | .573 | 62.9 | | NE. | Morning and afternoon cloudy; evening clear. |
| 11 | 29.728 | .153 | 73.7 | 2.7 | 17.0 | 20.0 | 59 | .649 | 66.4 | 0·709 | E. | Cloudy; afternoon and night, *rain.* |
| 12 | 29.588 | .139 | 77.7 | 3.0 | 15.0 | 10.0 | 63 | .717 | 69.3 | | (Var.) | Morning and afternoon cloudy; evening clear. |
| 13 | 29.699 | .111 | 81.3 | 3.7 | 8·0 | 5.0 | 75 | .908 | 76.4 | 0.073 | SW. | Cloudy; 11¾, A.M. *rain*, with thunder for an hour. |
| 14 | 29.777 | .078 | 85.3 | 4.0 | 15.0 | 5.0 | 48 | .697 | 68.5 | | S. | Morning cloudy; afternoon and evening clear. |
| 15 | 29.625 | .152 | 84.8 | 2.5 | 12.0 | — | 53 | .750 | 70.7 | | (Var.) | Cloudy. (*Instruments removed to 24th Ward.*) |
| 16 | 29.715 | .089 | 81.0 | 5.2 | 21.0 | 17.0 | 34 | .451 | 56.1 | | W. | Morning cloudy; afternoon and evening clear. |
| 17 | 29.648 | .067 | 85.3 | 4.3 | 30.0 | 42.0 | 36 | .595 | 63.9 | | SW. | Clear. |
| 18 | 29.512 | .135 | 89.0 | 3.7 | 24.0 | 31.0 | 31 | .555 | 61.9 | | SW. | Clear. *Thermometer highest* 100°. |
| 19 | 29.563 | .081 | 75.7 | 13.3 | 6.0 | 20.0 | 39 | .374 | 51.0 | | W. | Morning and evening clear; afternoon cloudy. |
| 20 | 29.670 | .107 | 73.3 | 3 7 | 19.0 | 19.0 | 31 | .314 | 46.3 | | NW. | Morning and evening clear; afternoon cloudy. |
| 21 | 29.739 | .070 | 75.7 | 2.3 | 26.0 | 42.0 | 35 | .441 | 55.5 | | NW. | Clear. *Thermometer lowest* 60°. |
| 22 | 29.800 | .063 | 77.3 | 1.7 | 27.0 | 42.0 | 31 | .414 | 53.8 | | N. | M. and ev. clear; aft. cloudy; 6 P.M. a few drops of *rain.* |
| 23 | 29.805 | .005 | 79.7 | 2.3 | 31.0 | 46.0 | 26 | .374 | 51.0 | | (Var.) | M. and ev. clear; aft. cloudy. *Therm. lowest* 60°. |
| 24 | 29.792 | .013 | 83.7 | 4.0 | 30.0 | 50 0 | 30 | .486 | 58.2 | | SW. | Clear. |
| 25 | 29.722 | .070 | 83.8 | 1.8 | 28.5 | 32 5 | 36 | .546 | 61.5 | | WSW. | Clear. |
| 26 | 29.703 | .042 | 86.0 | 2.8 | 33.0 | 40.0 | 37 | .624 | 65.3 | | WSW. | Clear. |
| 27 | 29.630 | .073 | 86.7 | 1.3 | 33.0 | 29.0 | 32 | .568 | 62.6 | | NW. | Morning and afternoon cloudy; evening clear. |
| 28 | 29.650 | .021 | 86.0 | 2.7 | 26.0 | 27.0 | 31 | .512 | 59.7 | | NNW. | Morning and evening clear; aft. cloudy; 6 P.M. high wind. |
| 29 | 29.685 | .063 | 80.7 | 5.3 | 18.0 | 12.0 | 57 | .689 | 68.2 | 0.073 | (Var.) | Cloudy; 8¼ to 11, A.M., *rain* at intervals. |
| 30 | 29.462 | .223 | 84.8 | 6.5 | 21.0 | 40.0 | 51 | 76 | 71.3 | | NW. | Cloudy. *Bar. lowest* 29.447. |
| 31 | 29.562 | .100 | 81.0 | 3.8 | 14.0 | 39.0 | 32 | .437 | 55.2 | | NE. | Morning and afternoon cloudy; evening clear. |
| Means for July, 1856 | 29.709 | .095 | 80.1 | 3.9 | 18.6 | 27.0 | 44 | .553 | 61.0 | 1.127 | S. 85° 22′ W. 25-100. | |
| Means for July, 6 yrs. | | | | | | | | | | | S. 77° 39¼′ W. 12-100. | |

